# The Natural Variation in Lifespans of Single Yeast Cells Is Related to Variation in Cell Size, Ribosomal Protein, and Division Time

**DOI:** 10.1371/journal.pone.0167394

**Published:** 2016-12-01

**Authors:** Georges E. Janssens, Liesbeth M. Veenhoff

**Affiliations:** European Research Institute for the Biology of Ageing, University of Groningen University Medical Centre Groningen, Groningen, The Netherlands; King’s College London, UNITED KINGDOM

## Abstract

There is a large variability in lifespans of individuals even if they are genetically identical and raised under the same environmental conditions. Our recent system wide study of replicative aging in baker’s yeast predicts that protein biogenesis is a driver of aging. Here, we address how the natural variation in replicative lifespan within wild-type populations of yeast cells correlates to three biogenesis-related parameters, namely cell size, ribosomal protein Rpl13A-GFP levels, and division times. Imaging wild type yeast cells in microfluidic devices we observe that in all cells and at all ages, the division times as well as the increase in cell size that single yeast undergo while aging negatively correlate to their lifespan. In the longer-lived cells Rpl13A-GFP levels also negatively correlate to lifespan. Interestingly however, at young ages in the population, ribosome concentration was lowest in the cells that increased the most in size and had shorter lifespans. The correlations between these molecular and cellular properties related to biogenesis and lifespan explain a small portion of the variation in lifespans of individual cells, consistent with the highly individual and multifactorial nature of aging.

## Introduction

The biology of aging is a multifaceted phenomenon resulting from a complex interaction between genes and the environment. Further complexity is added by the fact that even in laboratory models, individuals that are genetically identical and that are raised under the same environmental conditions, still have highly variable lifespans showing the typical sigmoidal lifespan curve at the population level [1–5].

*Saccharomyces cerevisiae*, or baker’s yeast, is a powerful model for studying the molecular mechanisms that define aging [6]. Baker’s yeast is a budding yeast, meaning that it undergoes mitosis by producing a daughter cell, termed a bud, on the mother cell. This asymmetric mode of cell division results in a somewhat smaller, age-reset daughter cell, and an increasingly enlarging and aging mother cell. This mother cell is able to undergo a limited number of divisions, termed the replicative lifespan (RLS) of the cell [7,8].

Recently, microfluidic techniques have been developed [9–12] which allow for constant and uninterrupted imaging of single yeast cells throughout their replicative lifespans. This now enables the exploration of factors related to the natural variation of lifespans in a population. In the current study we probe the correlative relationships between a cell’s replicative lifespan and the increase in size that a cell undergoes while aging, the ribosome concentration within the cell, and the division time of the cell.

Cell size, ribosomes, and cell division time have all been previously implicated in yeast replicative aging. The role of cell size is controversial. Cell size has both been suggested to be independent from replicative lifespan [13–17], and causal to it [18–20] and has also previously been studies in microfluidic devices [9–12,21–23]. Ribosomes, which are involved in cell size control [14], have been robustly shown to extend lifespan when knocked out in yeast and higher organisms [24–30], and have also been causally implicated in aging [31]. Cell division time has been found to be negatively correlated to lifespan when looking at the population level [15,32], and positively correlated to lifespan when assessing the mother cells separate from the population ([33] S7A Fig therein). While these observations relating cell size, ribosomes, and cell division time to lifespan have all revolved around mutant strains and altered environmental conditions, little has been explored regarding how they correlate to the natural variation of lifespans in a wild-type (WT) population, which our work now addresses.

Here, we compiled two large microfluidics-based datasets of 119 and 106 single cell life history recordings. For each replicative age of the cells, we assessed cell size increase and division time, which we found to always negatively correlate with lifespan. In the second dataset we used a GFP tagged protein of the large subunit of the ribosome, Rpl13A, to serve as a readout for ribosome concentration in the cells, and found this to have an age-dependent correlation to lifespan which is distinct early and late in the RLS. Our data shows that highly individual aging trajectories exist for single cells, and that the parameters of cell division time, levels of Rpl13A and cell size can be used to explain the variation in lifespans between individual cells.

## Materials and Methods

### Yeast strains and cultivation

*Saccharomyces cerevisiae* from the BY4742 background with a GFP-tagged protein [34], were cultivated in yeast nitrogen base medium, supplemented with 2% glucose and all amino acids except histidine. Strains were plated from frozen stocks and inoculated into liquid culture from plate, cultivated overnight and with dilutions to ensure exponential growth (10^7^ cells/ml) before loading the chips. This culture was diluted to 2–4 x 10^6^ cells/ml prior to loading onto the microfluidic chips to ensure optimal trapping of cells. The cells loaded in the chips are young as pre-culturing at mid exponential growth phase ensures an age distribution where the vast majority of cells are either newborn (age 0) or have budded only once or twice. Experiments numbered 1–6 (data in S1 File, Tables a and b) were performed in strains from the GFP-fusion collection [34] expressing C-terminal GFP-fusions of natively abundant cytosolic proteins, respectively: Rpl13A, Sod1, Hsp104, Rpl20A, Tps2, Hsp26. These were loaded into the microfluidic chip described in [9]. The data from all strains were combined after confirming that leaving out data from any one strain did not impact the average lifespan, cell size or cell cycle kinetics. The limited sample size per strain precludes a more detailed comparison between the strains. Experiments numbered 7–9 (data in S1 File, Tables c-e) were performed with Rpl13A-GFP loaded into the microfluidic chip described in [12].

### Microfluidics

Single yeast cells were imaged during their replicative lifespans on two different types of microfluidic dissection platforms, as described below.

Experiments 1–6 (data in S1 File, Tables a and b) were performed using the microfluidic device described in [9]. The platform was set-up and operated as described previously [35,36] with a flow rate of 3.4 μl/min, with one alteration: the side channel was omitted from chip construction and replaced with an outlet hole in the main channel of the chip above the pillar section, which served the same purpose as the side channel. The platform was loaded onto a commercial Nikon (Eclipse Ti-E equipped with autofocus capabilities, solid state illumination (pE2-CoolLed, 15% intensity) and a CFI Plan Apo 60×/1.40 oil objective) or Zeiss (Axio Observer.Z1 equipped with Definite Focus and solid state illumination (Colibri, 25% intensity) using a Plan Apo 63×/1.40 oil objective) microscope and cells were imaged every 20 minutes for ~120 hours, the time required to view the full replicative lifespan of the starting population. Exposure of cells in the fluorescent channel was adjusted for each strain with short exposure times (70–300 ms on Nikon and 150 – 300ms on Zeiss), on each imaged frame. Experiment 6 only used bright-field imaging.

Experiments 7–9 (data in S1 File, Tables c-e) were performed using the microfluidic device presented in [12]. The platform was set-up and operated as described therein with a flow rate of 2.9 μl/min (divided over two syringes). The platform was loaded onto a commercial DeltaVision microscope (Applied Precision (GE), equipped with autofocus capabilities, solid state illumination Plan Apo Olympus 60×/1.42 oil objective) and cells were imaged every 20 minutes for ~120 hours, the time required to view the full replicative lifespan of the starting population. Exposure of cells in the fluorescent channel was with low intensity (10% of LED) with short exposure times (100 ms), on each imaged frame.

### Analysis of published data

Supplemental data from Huberts *et al*. 2014 [21] was downloaded and lifespan data from BY4741 cells grown using the microfluidic platform in 2% glucose was used to evaluate lifespan curves generated with and without censored data.

### Dataset collection

Cell sizes were outlined using the fluorescent channel of the cell images, with FIJI (ImageJ) software [37], using preinstalled plugins and image processing functions therein. Briefly, the channel brightness was increased, median smoothing was applied (radius of 2), background was subtracted, the channel was converted to binary, and watershed, outline, and skeletonize algorithms were applied, producing final outlines of the cells. Using these cell outlines, cell areas were measured at every frame prior to a budding event. In cases in which the data outlining macro failed to produce the outline of the cell, manual outlining of the cells was performed. In one case, experiment 6, visible-light spectrum images were collected and manual outlining was performed on all cells.

Cells were included in the analysis which had their full lifespans observed (i.e. were not washed out during the experiment). In several instances, a daughter cell coming from the original cohort born early in the experiment had a full lifespan observed, and was included in the analysis. In rare instances, cells present in the original cohort died without division, or produced only one bud prior to death, and were omitted from the data collecting process. In general these cells underwent a large growth prior to death.

Only the first G1 observed in the microfluidic chip (i.e. the cell present in an unbudded state, directly prior to a budding event) was taken as the first measurement. The size of the cell at death was not included in the analysis (not present in data Tables). Cell sizes were converted from pixel values to μm^2^ units. The cell sizes in μm^2^ of the first dataset analyzed (119 cells total, experiments 1–6) are presented in data in S1 File, Table a with corresponding time measurements in S1 File Table b, starting from 0 hours relative to the first G1 observed. μm^2^ sizes of the second dataset (106 cells total, experiments 7–9) are presented in data in S1 File, Table c with corresponding time measurements in S1 File Table d, starting from 0 hours relative to the first G1 observed. In the second dataset (tracking ribosome abundance, Rpl13A, experiments 7–9), the cells were outlined and the average fluorescence intensity was corrected for background fluorescence present in the same image frame. Average fluorescence intensity data of Rpl13A for each cell at each replicative age is shown in S1 File Table e.

### Analysis of compiled growth data

Analysis and visualization of the data was performed using the R scripting environment [38]. The longitudinal profiles (cell size increase and fluorescence intensity) of individual cells, from the first to last observed G1, were fitted with cubic smoothing splines. Spline smoothing fit of the data was performed using the preinstalled smooth.spline function in R using a smoothing parameter of 0.5. Prior to fitting of the data, starting and ending cell sizes were assessed for their correlations to lifespan. The fitted data was used for all other analyses. Determination of the Senescence Entry Point (SEP) [11] was done by visually inspecting the occurrence of an ‘elbow’ in the curve of cell cycle duration at each age (i.e. the farthest point from a line drawn between the first to last ages of a cell in the plot). Lifespan data was assessed using the ‘Survival’ package in R [39], with Kaplan-Meier estimated survival curves compared to each other using the log-rank test. Population distributions were assessed with the Wilcoxon (also known as Mann-Whitney U) statistic using the preinstalled wilcox.test function in R. Where appropriate, differences in data were considered to be significantly different with a p value less than 0.05. Data visualization was done using the R package ‘gplots’ [40].

## Results

### A dataset of life histories of single cells for lifespan analysis

**Fig 1:**
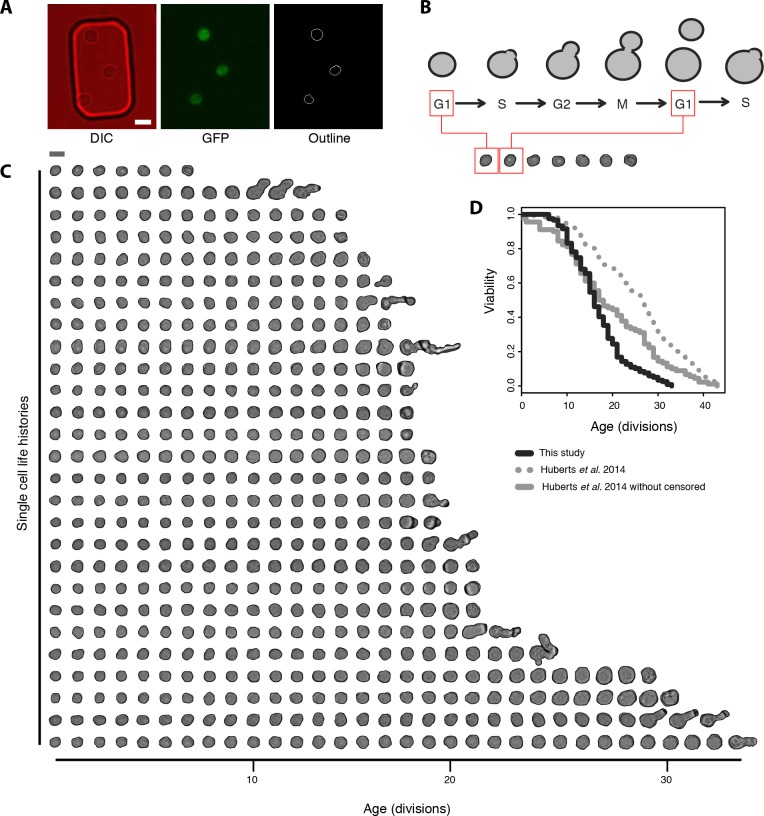
Microfluidics dataset of cell size and division time measurements in yeast replicative aging. (A) Example of yeast cells in the microfluidic chip (left panel, DIC) expressing endogenous protein tagged with GFP (middle panel, GFP) used for cell outlining to measure area of cells (right panel, Outline). Scale bar represents 5 μm. (B) Schematic representation showing the time point at which the cell area was outlined: just prior to a budding event, i.e. in G1 phase of the cell cycle. (C) Visualization of a subset of the dataset where each DIC image represents the cell size directly prior to a budding event. The last cell shown in each life history row is an image of the cell’s death (not included in analysis). Scale bar 5 μm. (D) Comparison of lifespan curves of BY strains grown on 2%-glucose obtained from the same microfluidic device. The black line represents data from this study, and the grey line represents the data from [21]. For the solid lines only cells that were observed throughout their entire lifespan until death were included in the analysis (this study: median RLS of 16, 119 cells; Huberts *et al*. 2014 [21]: median RLS of 17.5, 90 cells). The grey dashed line represents data from [21] and yields a median RLS of 26 (grey dashed line, 90 cells with full lifespan, 810 cells included as censored).

In order to generate a dataset of cell size changes occurring in aging yeast at the single cell level, we took advantage of microfluidic based time-lapse microscopy capable of following yeast throughout their entire lifespans [9,35,36]. The method traps an initial cohort of young cells under pillars of soft silicon material (Fig 1A, left panel), while providing a fresh flow of medium that washes away emerging daughter cells. Cells were grown in YNB glucose medium at 30 degrees and images were taken every 20 minutes. Outlining the cell circumference and measuring its area determined the cell size. Each cell cycle measurements were taken just prior to a bud emerging, i.e. at the G1 phase of the cell cycle when the mother is present in an unbudded state (see Fig 1B for schematic representation of when cells were outlined in the cell cycle).

Fig 1C shows DIC images of 27 manually outlined cells throughout their replicative lifespans. The first image in each life history row represents the cells at the first G1 after loading the chip, directly prior to the first fully observed budding event. The last image is of the cell’s death, defined as a sudden shrinking of the cell and cessation of budding. These dead cell images were omitted from further analysis. In order to generate a larger dataset of age-dependent cell sizes, we used strains expressing Green Fluorescent Protein (GFP) tagged cytosolic proteins. The GFP signal was used with image processing to facilitate the outlining procedure (see Fig 1A, left panel for cells trapped in the microfluidic chip, middle panel for GFP fluorescence and right panel for image-processing aided outlining). Each cell outline was manually curated for accuracy, and in total, from six microfluidic experiments we compiled a dataset of 119 cells for which we had 1994 manually verified data entries of the replicative age and time dependent cell sizes.

As a first assessment of the compiled dataset, we determined the lifespan curve and median RLS of the cells. Here, we found the expected sigmoidal-shaped survival distribution, with a median lifespan of 16 divisions (Fig 1D, black line). This median lifespan is based on cells that remain in the field of view of the microscope until their deaths are observed, and is thus shorter that the true median life span of the cells. To ensure our cells have a normal rate of aging we compare our data to previously published data obtained with the same microfluidic device [21]. *Huberts et al*. 2014 calculated a median life span of ~26 divisions (Fig 1D, dotted grey line), when including the cells that were flushed out with the flow of medium during the experiment (censored cells); a lifespan similar to what is observed with the microdissection method. Re-analyzing the data from [21] while only including cells that were present until their deaths were observed (Fig 1D, grey line), we find a median lifespan of 17.5 divisions, similar to our median life span of 16. We thus conclude that our microfluidics dataset that covers a wide range of lifespans, from 6 to 33 replications, can be used in order to explore the natural variation in lifespans present within a population.

### Cell size profiles in single aging yeast and the assessment of starting and end sizes relative to replicative lifespan

**Fig 2:**
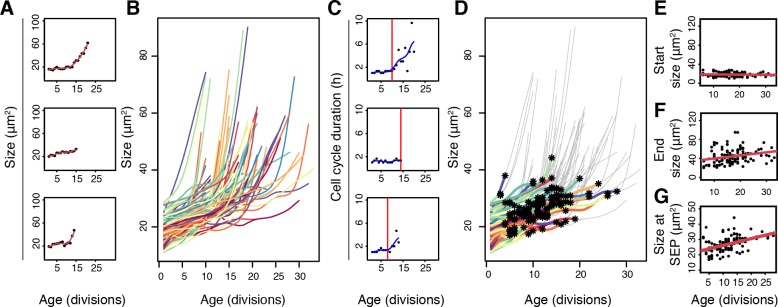
Cell size profiles in single aging yeast and the assessment of size relative to replicative lifespan and Senescence Entry Point. (A) Spline fitted data of cell size measurements of 3 single cells. Size reflects cross-sectional area (μm^2^) of the cell in the microfluidic chip. (B) Cell sizes of the 119 cells in the dataset throughout their replicative lifespans shows cell size increase with each division and variation present within the population. (C). Illustration of single cell profiles and the Senescence Entry Point (SEP) (red line) of cells shown in A. (D) The entire population of cells as presented in B, with data entries plotted in color up until the SEP (asterix), and plotted in grey thereafter. (E) Starting sizes of cells compared to their replicative lifespan (Pearson correlation -0.047). Median of cross-sectional size of population corresponds to 17.65 μm^2^, approximately equal to a diameter of 4.74 μm. (F) End sizes of cells compared to their end replicative lifespan (Pearson correlation 0.255). (G) Size of cells at SEP compared to their replicative age at the SEP (Pearson 0.4479)

Cell size has long been described to increase throughout yeast replicative aging ([41] and also clear from Fig 1C), the dynamics of which we aimed to determine at the single cell level complementing previous studies using microfluidic devices [9–12,21–23]. To resolve cell size profiles of each individual cell in our data, pixel areas were converted to μm^2^ areas (S1 File Table a, with corresponding division times in S1 File Table b), and to accommodate for measurement errors, we fitted the longitudinal datasets with a cubic smoothing spline that we resampled at each replicative age (methods and Fig 2A and S1 Fig). This resulted in 119 single cell profiles of size and age (Fig 2B). As has been previously reported [11], cells can enter a ‘Senescence Entry Point’ (SEP) late in life. To analyzed our data within the context of the SEP, we plotted the cell cycle duration at each replicative age for each cell and determined the point of inflection at which cell cycle duration drastically changed (Fig 1C). In line with previous reports [11], we see that the slowed cell cycle is indeed when the greatest size increase occurs (Fig 1D and S1 Fig).

Having compiled a dataset of cell sizes for each replicative age of single cells, we first aimed to see whether the starting size of the cells reflected their lifespan potential and whether a critical cell size may be limiting the cell’s lifespans in this WT population. Using our dataset of cell sizes coming from a WT population, we found no clear correlation (Pearson correlation -0.047) to exist between the starting size of the cell (size directly prior to budding) and its replicative lifespan (Fig 2E). Furthermore, we found no evidence showing that cells end their lives (Fig 2F) or enter the SEP (Fig 2G) at a fixed size, rather we observe small positive correlations for both. In conclusion, our data argues against the ‘hypertrophy’ model and the possibility that a critical cell size limits lifespan, in WT cells (i.e. see [16,17,19]).

### Increases in cell size at even early ages reflect replicative lifespan potential in yeast

**Fig 3:**
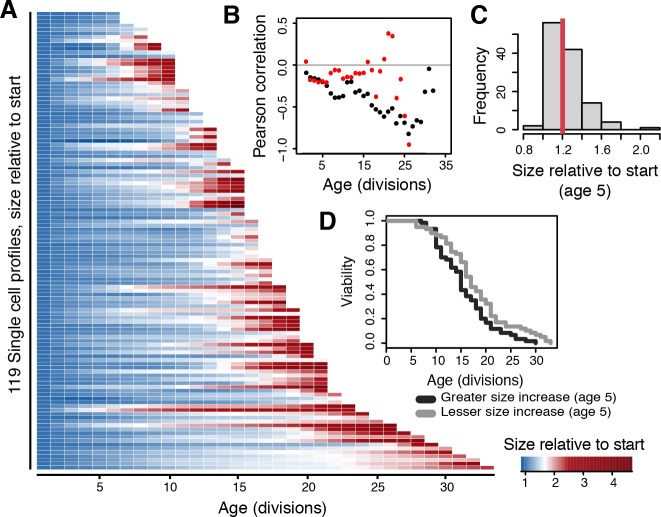
Early life increase in cell size reflects lifespan potential of single cells. (A) The amount each cell has increased in size at any given age. Cell size increase is represented as color intensities, measured in terms of fold increase relative to starting size. (B) Pearson correlations comparing the observed population lifespans to cell size increase at each respective age in the complete dataset (black dots) and in all pre-SEP datapoints (red dots). (C) The frequency distribution of amounts of cell size increase in the population at age 5. Red line indicates the median, an increase of 1.2 times the starting size. (D) Lifespan curves of cells that have increased less in size (grey line, 59 cells, median RLS of 17) or more in size (black line, 60 cells, median RLS of 15) than the median of the population at age 5 (p-value = 1.7 x 10^−2^).

We next asked if the amount of increase in size a cell had undergone *at any particular age* related to its lifespan. To do so, we normalized all cell areas to each cell’s first G1 cell size. This resulted in all profiles having a normalized starting size of ‘1’, with subsequent time points reflecting the cell size increase. Representing these values as color intensities highlights the variation in cell size increase existing even at early ages in the population (Fig 3A).

We addressed how early life variation of cell size change related to the variation in lifespan. Here we found even at younger ages (age 5) a small (0.2) negative correlation to exist between lifespan and the amount a cell had increased in size at each respective age (Fig 3B, black dots). The correlation becomes stronger at later ages but this is mostly due to cells undergoing a much greater increase in size directly prior to death [9] and after the ‘Senescence Entry Point’ (SEP) [11]; indeed when excluding from the analysis the post-SEP data points this decrease is not observed (Fig 3B, red dots).

To assess the importance of the early life small negative correlation, we selected a young age time point (5 divisions, a time point prior to any occurrence of mortality in the dataset), and split the population by the median value that cells had increased in size at that age (1.2 times their starting size). The resulting two populations thus reflected cells increasing more in cell size with age than the median, or less (Fig 3C). An analysis of the lifespans of these two populations revealed a significant difference in survival probability, with the cells increasing less in size living 13% longer when comparing the medians of the two groups (Fig 3D). We conclude that the amount a cell increases in size per division is related to the end lifespan it will attain, contributing to variation of lifespan in the population, and that this trend is visible even at early replicative ages.

### A dataset reflecting ribosome concentrations present in single cells throughout aging

Following from the observation that the increase in size an aging cell undergoes has an inverse correlation with its lifespan, we next aimed to see if ribosomes, which are in part responsible for biomass production and cell size control [14,42,43], could serve as a molecular biomarker associated to the cell’s lifespan. We used a component of the large ribosomal subunit, Rpl13A, tagged with a C-terminal GFP, to serve as a reporter reflecting ribosome abundances in the cells. RPL13A was chosen as it is exposed to the surface of the ribosome [44] and hence tagging is least expected to impact assembly or function. Indeed, C-terminally tagged versions of RPL13A were shown to have normal growth characteristics [45] and immunoprecipitation experiments were indicative of proper incorporation of RPL13A-GFP in ribosomes (S2 Fig). In three experiments using an alternative microfluidic device [12] we generated a second dataset comprising life histories for 106 cells. In these microfluidic devices the cells are trapped in cages that have gaps through which budding occurs, leading to removal of daughter cells by the flow of media. Collecting and processing the cell images as was performed with our first dataset (Fig 1A and Methods), we again generated manually curated cell size information for each division in the replicative lifespan of the cells (S1 File Tables c and d, respectively). We note that in this second dataset we find similar correlations of single cell lifespans relative to ending cell size, starting cell size and increase in size per division (S3 Fig panels DEF).

**Fig 4:**
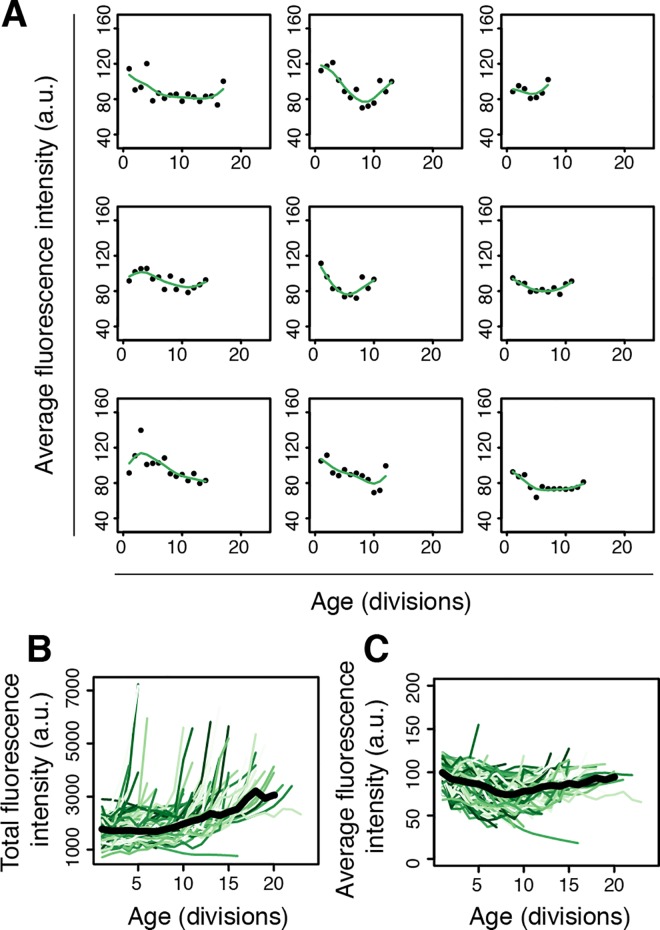
Ribosomal protein Rpl13A-GFP levels in single cells throughout aging. (A) Spline fitted data of nine single cell profiles of the average fluorescence intensity levels of Rpl13A-GFP present in the cell with age. (B) Assessment of total Rpl13A-GFP levels with age by tracking the total fluorescence intensity shows an overall total increase in Rpl13A-GFP with age. Dark line is median of population. (C) Assessment of Rpl13A-GFP concentration with age by tracking average fluorescence intensity shows a constant or decreasing concentration of Rpl13A-GFP within the cell with age. Dark line is median of population.

Using the average intensity from the fluorescent channel for the outlined cells, we generated abundance information of Rpl13A present in the cells at each replicative age (S1 File Table e). As with the first dataset, we fitted the longitudinal cell size and average fluorescence intensity values with cubic smoothing splines that we resampled at each replicative age (Fig 4A, S3 Fig panel A). Plotting the total fluorescence intensity (calculated from the size in μm^2^ multiplied by the average fluorescence intensity) for each age gave an indication of the total amount of Rpl13A present in each cell throughout its lifespan (Fig 4B), and at the population level showed an increase to occur with age (S3 Fig panel B). This is in agreement with literature suggesting an increase of ribosomes to occur with aging in yeast [31,46]. Plotting the average intensity on its own however indicated that the concentration of ribosomes within the enlarging cell tends to slightly decrease with age (Fig 4C, S3 Fig panel C). In order to assess ribosome abundance in aging cells in a unit independent of cell size, we used the concentration data of Rpl13A (average fluorescence intensity, Fig 4C, rather than total abundance, Fig 4B) in cells for subsequent analysis and comparisons relative to lifespans.

### Ribosome concentrations in single cells are an age-dependent marker for lifespan

**Fig 5:**
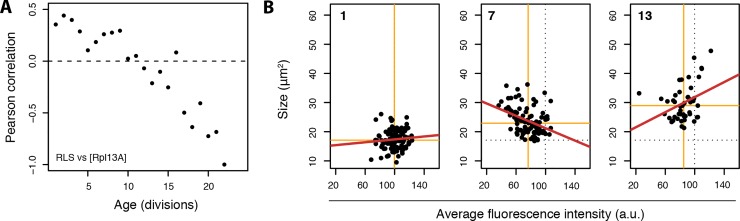
Ribosomal protein Rpl13A-GFP concentrations in single cells reflect replicative lifespan potential in yeast. (A) Pearson correlation comparing Rpl13A-GFP concentration of cells to their end lifespans attained. Dashed line is 0 correlation reference. At younger age a positive association exists, where a higher Rpl13A-GFP concentration is indicative of a longer lifespan. This association steadily deceases and at older age the inverse is true, lower Rpl13A-GFP concentration is indicative of higher likeliness to remain alive. (B) Scatterplot of Rpl13A-GFP concentration compared to cell size at representative ages (top left number in each panel). Orange solid lines are median values, black dashed lines are median values at age 1, red solid lines are linear regressions. Graphs indicate that cells increasing rapidly in size undergo dilution of Rpl13A-GFP (i.e. larger cells have lower concentrations, middle panel, age 7), and therefore that the positive association of ribosome concentration to lifespan observed at young ages in (A) reflects the fact that cells with higher ribosome concentrations have increased less in size. Reversal of this trend later in age (right panel, age 13) implies that the inverse correlation of ribosome concentration to lifespan present at later ages also relates to cell size. See S4 Fig panel A for plots of more ages.

Having compiled a dataset of Rpl13A-GFP levels in aging cells, we next asked how this related to the variation of lifespans present in the population. Previous work has shown that many ribosome deletions, including Rpl13A, result in increased lifespan for yeast [25], and that the translation machinery such as the ribosomes are implicated as a driving force in the aging process [31]. Therefore, we expected a negative correlation would exist between ribosome concentration in single cells and lifespan, such that at any given age, cells with lower concentrations of ribosomes would be more likely to be the longer-lived cells in the population. To address this hypothesis, we performed correlation analysis of ribosome concentrations and lifespans of cells in the population, for each replicative age (Fig 5A).

Examining these correlations, we found a negative correlation to exist, albeit only clearly at older ages (Fig 5A). This indicated that at older ages, after more than half of the population has died due to aging and cells that are post the SEP are more prevalent in the population, levels of ribosomes are inversely related to survival probability and begin to reflect the remaining lifespan potential of cells. Interestingly, at earlier ages, where post-SEP cells are not yet affecting the population averages, we found the opposite correlation to exist, namely, cells with higher concentrations of ribosomes tended to have longer replicative lifespans (Fig 5A). Although this second observation was counterintuitive, it nonetheless highlighted that also at younger ages, levels of ribosomes are related to survival probability, and therefore reflect the replicative lifespan potential of the cells.

To better understand how a positive correlation could exist at younger ages between ribosome concentrations and lifespans, we plotted ribosome concentrations versus cell size for cells at different ages (Fig 5B and S4 Fig panel A). Here, we found that at young ages, the larger cells are those with lower concentrations of ribosomes (Fig 5B, middle panel, age 7), likely resulting from a dilution effect in the cytosol occurring in the enlarged cells. This indicated that the positive correlation between ribosome and lifespan present at young ages is a reflection of the rate of cell size increase. Namely, cells that increase more in size have diluted concentrations of ribosomes, and a shorter lifespan, while cells that have greater ribosome concentrations are in fact smaller, and live longer. Therefore, ribosome concentrations at younger ages mainly reflect cell size increase (Fig 5) which itself strongly correlates to lifespan potential (Fig 3). An explanation why rapidly enlarging cells tend to live shorter lives may be that a dilution of certain molecular constituents is occurring within them.

In line with the above observations that at young ages ribosome concentrations and cell size are inversely correlated, at older ages, ribosome concentrations begin to reflect cell size and become positively correlated (Fig 5B, right panel, age 13). This indicates that the concentration of ribosomes becomes more relevant as a parameter reflecting cell size increase, and therefore lifespan, only once the short-lived cells die out of the population. Indeed, performing the correlation analysis (Fig 5A) on only longer lived cells in the population, reveals the negative correlation we expected to see between ribosome concentration and lifespan at all ages (S4 Fig panel B).

### Cells that live longer and increase less in size divide faster

**Fig 6:**
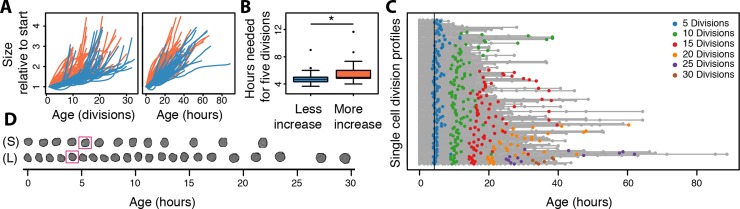
Cells that live longer and increase less in size progress faster through the cell cycle. (A) Cell size change of cells that increase less (blue) or more (orange) in size early in life as compared to the median change in cell size at age 5 replications; same populations as in 3D and E. Comparing cell size increase to age in replications (left panel) or time (right panel) highlights that the cells increasing less in size per division also increase less in size in time. (B) Distribution in the same two subpopulations for the hours it takes for cells to reach age five (replications). Comparing the cells that increase less (blue) or more (orange) in size, shows that the longer living cells that increase less in size (blue) require less hours to reach the same replicative age, and therefore progress faster through the cell cycle (p-value = 3.2 x 10^−5^). (C) Single cell division profiles looking at the time at which each cell divides to further illustrate that longer lived cells progress more rapidly through the cell cycle. Grey lines indicate single cells organized by their replicative lifespan, from shortest (top) to longest (bottom) lived (same order of cells as Fig 3A). Colored dots highlight specific divisions of a cell (see legend), grey dots all others. Comparing black vertical line to blue dots (age 5) illustrates that longer-lived cells (bottom of graph) reach this age sooner in time (i.e. progress faster through the cell cycle), and that shorter living cells (top of graph) need more time (i.e. progress slower through the cell cycle). See S4 Fig panel C for replicate validation. (D) Illustration using two cell profiles selected from Fig 1C showing that longer-lived cells (L) in general reach a replicative age of 5 (magenta square) more rapidly than do shorter-lived cells (S).

Having information on the duration of each of the cell’s divisions, we asked how cell size increase, replicative lifespan, and division time are related to one another. To do so, we returned to our initial dataset used to compare cell sizes and lifespans (119 cells, Figs 1–3). Using the same two subgroups of cells that either increased more or less in size per division relative to the median of the population at age 5 (Fig 3C and 3D, Fig 6A left panel), we plotted their cell size increase relative to the time they were alive (Fig 6A right panel). Here, we saw a trend that the cells increasing less in size per division (Fig 6A, left panel, blue group), also had more rapid division times (Fig 6A right panel, blue group). Consistent with [9–12] the cell divisions become slower towards the end (Fig 6A left panel). Interestingly, while the cell size relative to start varied greatly from cell to cell, the rate of increase per time seemed to remain rather constant throughout the life of a single cell, suggesting a cell specific setting for the rate at which a cell increases in size (Fig 6A right panel). Seeing these observations, we asked what the difference was between the division times in the two populations, i.e. of cells either increasing more (orange group), or less (blue group), in size relative to the median of the population at (replicative) age 5 (derived from Fig 3C). We assessed the amount of time these two groups needed to reach age 5, plotting boxplots of their distributions (Fig 6B). Indeed, we found that the cells that increase less in size per division, which tend to live longer, also divided more rapidly (Fig 6B).

Finally, we plotted the single cell division profiles in time, ordered by their replicative lifespans (Fig 6C). Here, we confirmed that cells that live longer (i.e. at the bottom of the graph) indeed appear to reach an age of 5 divisions (blue dots) earlier than shorter lived cells (present at the top of the graph) (Fig 6C and confirmed in the replicate dataset, S4 Fig panel C). This observation seems to continue the farther in a cell’s replicative lifespan one looks. For instance, at 20 divisions (orange dots), the cells with longer replicative lifespans have reached this age sooner (Fig 6C), which is consistent with observations that cells that are just prior to death slow in their division times (reviewed in [6]). Indeed, typical life histories illustrate that even at a young age, replicative lifespan and division time are intimately connected (Fig 6C), namely, compared to shorter living cells (S), longer living cells (L) divide faster, and ultimately have longer replicative lifespans (Fig 6D), contributing to the natural variation of lifespans in the population.

## Discussion

**Fig 7:**
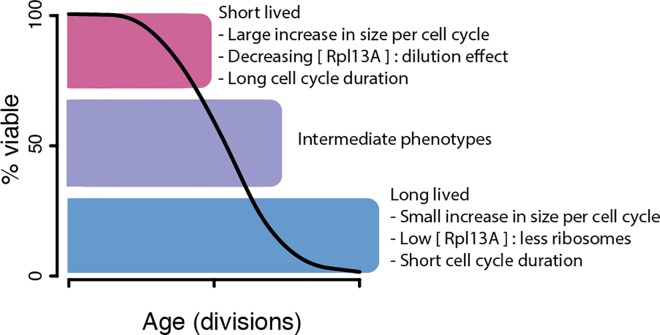
Summary model of observations. Cells that die early in the population (magenta block at top of lifespan curve) are generally having a large increase in size per division, decreasing Rpl13A concentrations and longer cell cycle durations. The decreasing Rpl13A concentration may be due to dilution of the cytosol occurring in the enlarging cells. Meanwhile, long-lived cells in the population (blue block at bottom of lifespan curve) are characterized by having a small increase in size per division, a short cell cycle durations and a low concentrations of Rpl13A (relative to other cells in the population after the short-lived cells have died). Intermediate phenotypes related to cell size increase, ribosome concentration, and division times occur in cells that are neither short nor long lived in the population (purple block in the lifespan curve).

Our findings have highlighted the association of cell size increase, ribosome concentrations, and cell division time to lifespan (Fig 7). Furthermore, while previous studies have operated at the population level or made comparisons between mutant strains or treatments, we have looked within a WT population at detailed life histories of single cells to identify factors contributing to the natural variation of lifespans. As illustrated in our graphic representation of the data (Fig 1C), each cell lives a unique life. We measured the increase in cell size per division (Fig 2A), ribosome levels (Fig 4A), and division times (Fig 6C) and found that even at early ages these parameters are predictive of lifespan potentials. Specifically, we found that cells that increase less in size per replication tend to live longer, ribosome concentrations are predictive of remaining lifespan at young and old ages albeit in opposing manners, and that cells living longer and increasing less in size tend to progress more rapidly through their cell cycle (Fig 7). As is to be expected for a multifactorial and stochastic process like aging, the correlations reported here are modest and only explain a fraction of the variation. Below we discuss the significance and implications of these findings in the context of literature.

### Cells increasing less in size per division live longer

Time-lapse microscopy and microfluidics have allowed us to collect a dataset of aging mother cells. We note that the first cell size in our analysis corresponds to a cell after it has undergone the initial cell size increase that occurs in virgin daughters before it commits to cellular division at the Start checkpoint. Although with this we could not consider cell birth-size or first G1 duration, which have been shown to be related to lifespan [15], it ensured that our measurements were independent of the cell cycle phase of the cells when loaded in the microfluidic chip. With this, we found no clear correlation with starting size and lifespan (Fig 2E). Furthermore, we observe that cells end their lifespans at a variety of different sizes (Fig 2B). This is in strong contrast to the ‘hypertrophy’ model of aging, which claims that a maximal size limits lifespan of the yeast [18–20], and is in agreement with molecular based views of causes of aging [16,17]. We did however find that already at early ages the increase in size per division shows a negative correlation with lifespan, which can be used to distinguish longer and shorter living cells in a population (Fig 3D).

### Ribosome concentration is predictive of remaining lifespan at young and old ages

Spurred by our observations relating cell size increase and lifespan, we aimed to see if ribosomes, which have been linked to cellular growth and cell size control [14,42,43], may serve as a molecular biomarker of aging in yeast. We found that ribosome concentration indeed reflected lifespan potential of yeast, both at young (positive Pearson correlation existing of ~0.4) and old (negative Pearson correlation existing of ~-0.6) replicative ages. The negative correlation observed at older ages is in line with findings that mutants with ribosome deficiencies live longer [25], and that translation machinery can be a driving force in aging [31]. The positive correlation observed at younger ages is likely due to rapidly enlarging cells having lower concentrations of ribosomes.

The distinct correlations between Rpl13A-levels and RLS at young and old ages in the population suggests that cells in the population may be dying due to different causes. For example, cells dying early may have undergone an extreme dilution of molecular constituents which is incompatible with life, while cells dying later may have died due to causes related to protein translation and homeostasis [47]. Indeed, prior work with microfluidic methods have found that cells die in various forms [9,10,22], indicating that heterogeneity is present in the mode and mechanism of death of the cells. Our work has served to illustrate this further. We have shown how a fluorescent-tagged ribosomal protein may serve as a biomarker of aging in yeast to identify remaining lifespan at young and old ages, albeit for different reasons in these young and old cells.

### Cells that live longer and increase less in size are dividing faster

We observed that cells that live longer and increase less in size per division tend to progress more rapidly through their cell cycle, compared to shorter-lived cells that increase more in size in the same population of WT cells (Fig 6 and S4 Fig panel C). Our study has quantified the cell cycle of isolated WT mother cells, as distinguished from proliferating populations (dividing mothers and daughters) of mutant strains, which much of the previous literature has been built upon. This is a noteworthy distinction. For example, comparing proliferating populations of mutant strains [15,32], a longer G1 phase was linked to longevity. However, since in a proliferating population at least half of the cells are newborn daughters that need time to reach a critical size before beginning their cell divisions, these studies have essentially demonstrated how cell cycle parameters of daughters correlate to the replicative capacity of mothers. This is distinct from our studies on cell cycle parameters of mother cells in relation to lifespan. It would be interesting to connect the observations in the future, for which we would need to know how the cell cycle parameters of a daughter cells depends on those of the mother cell, but at present a direct comparison to previous studies is not possible.

Our data has shown that cells increase in size at a relatively stable and constant trajectory *in time* (Fig 6A, right), as compared to the increase the aging cell undergoes *per division* (Fig 6A, left). This indicates that a replicatively aging cell’s increase of size is more dependent on time rather than its divisions, and that the large increases in sizes reported to occur late in replicative age ([9] and seen in Figs 2B and 3A) are largely due to the prolonged cell cycle that accompanies this size increase. Therefore, cells able to complete a cell cycle more rapidly in time will incur less cellular size increase per division (Fig 6D). This implies that, beyond a cell’s first division, division frequency of an aging mother cell may not depend on its size [48], and may depend on other factors, if any. In our data, we find that the mother cell’s division time follows different correlations to ribosome concentration at young and old ages, suggesting that an age-dependent relationship may exist in the factor(s) determining the mother’s cell cycle length. Surely, further work to identify factors affecting division frequency of aging mother cells will result in the identification of factors that can influence the replicative lifespan of the cells as well.

## Supporting Information

S1 Fig Cell size profiles in single aging yeast and Senescence Entry Point (SEP). (A) Spline fitted data of cell size measurements of six single cells. Size reflects cross-sectional area (μm^2^) of the cell in the microfluidic chip. Red star indicates where cells enter SEP as derived from B. (B) Single cell profiles of the cell cycle duration at each replicative age and the SEP (red line) of the same cells as in A.
(TIF)

S2 Fig Immunoprecipitation using anti-GFP antibody of RPL13A-GFP. The ribosomal proteins, RPL15, Rpl13A and are detected by western blot in the immune-precipitated fractions. Coomassie brilliant blue stained gel shows total proteins in input, unbound and elution (IP) fractions. An immunoprecipitation of RPL20-GFP under identical conditions is shown as a comparison. Whole cell extracts were prepared from mid exponential cultures using a FastPrep-24 Instrument (MP Biomedicals, Santa Ana, CA, USA), antibodies were from Abcam (Cambridge UK; ab98211; ab130992; ab90874), GFP-TRAP_A agarose beads were obtained from ChromoTek (Planegg-Martinsried, Germany) and used according to inductions by manufacturer.
(TIF)

S3 Fig A second dataset of single cell life histories, with Rpl13A concentration changes. (A) Cell sizes same as Fig 2B but for the second dataset. (B) Same as Fig 4B but showing the fold change of the total fluorescence intensity of Rpl13A in the cell with age, indicating an increase in fold. Dark line is median. (C) Same as Fig 4C but showing the fold change of average intensity (concentration) of Rpl13A in the cell with age. Dark line is median. (DE) Same as Fig 2E and 2F, respectivelyA small negative correlation is found between starting cell size and lifespan (Pearson correlation -0.203 versus -0.047 in the first dataset). A small positive correlation is found between ending size and lifespan (Pearson correlation 0.101 versus 0.255 in the first dataset). (F) Same as 3B, showing a small negative correlation of lifespan compared to cell size increase.
(TIF)

S4 Fig A second dataset of single cell life histories, cell size, ribosome concentrations, cell cycle times, and lifespans. (A) Illustrates relationship between ribosome concentration and cell size throughout aging as does Fig 5B but for additional ages (numbers in top left of panels). (B) Same as Fig 5A (black dots, with stars), but illustrating the correlation of ribosome concentration to lifespan, when only considering cells in the population that lived at least to age 8, 12, 16, or 18. Illustrates that as the short lived cells are progressively removed from the analysis, the negative correlation of ribosome to lifespan becomes more prominent. (C) Same as Fig 6C but for second dataset. Confirms that longer-living cells (RLS, bottom of graph) divide more rapidly, i.e. reaching age 5 (blue dots) sooner in time than shorter-lived cells (RLS, top of graph).
(TIF)

S1 File Data on cell sizes, average fluorescence intensity and corresponding time measurements. Table a: The cell sizes in μm2 of the first dataset analyzed (119 cells total, experiments 1–6). Table b: Corresponding time measurements to cells in Table a. Table c: The cell sizes in μm2 sizes of the second dataset (106 cells total, experiments 7–9). Table d: Corresponding time measurements to cells in Table c. Table e: Average fluorescence intensity data of Rpl13A-GFP for each cell at each replicative age from the second dataset (106 cells total, experiments 7–9).
(XLSX)
