# Supplementary figures and images for: The Natural Variation in Lifespans of Single Yeast Cells Is Related to Variation in Cell Size, Ribosomal Protein, and Division Time
Source: PLoS One. 2016 Dec 1;11(12):e0167394. doi: 10.1371/journal.pone.0167394 (PMC5132237; doi:10.1371/journal.pone.0167394)

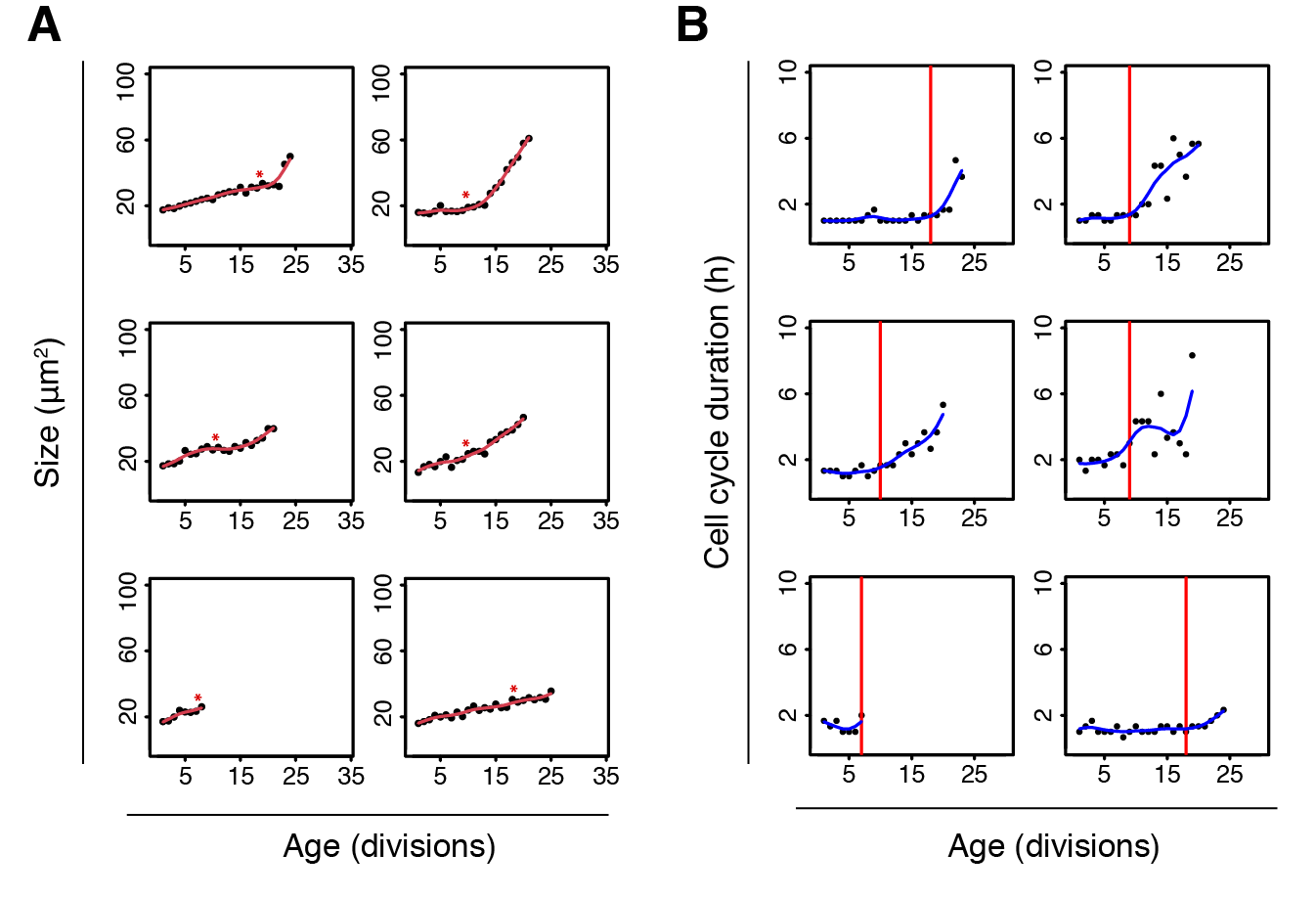

Supplement: S1 Fig — (A) Spline fitted data of cell size measurements of six single cells. Size reflects cross-sectional area (μm2) of the cell in the microfluidic chip. Red star indicates where cells enter SEP as derived from B. (B) Single cell profiles of the cell cycle duration at each replicative age and the SEP (red line) of the same cells as in A. (TIF) [file pone.0167394.s001.tif]

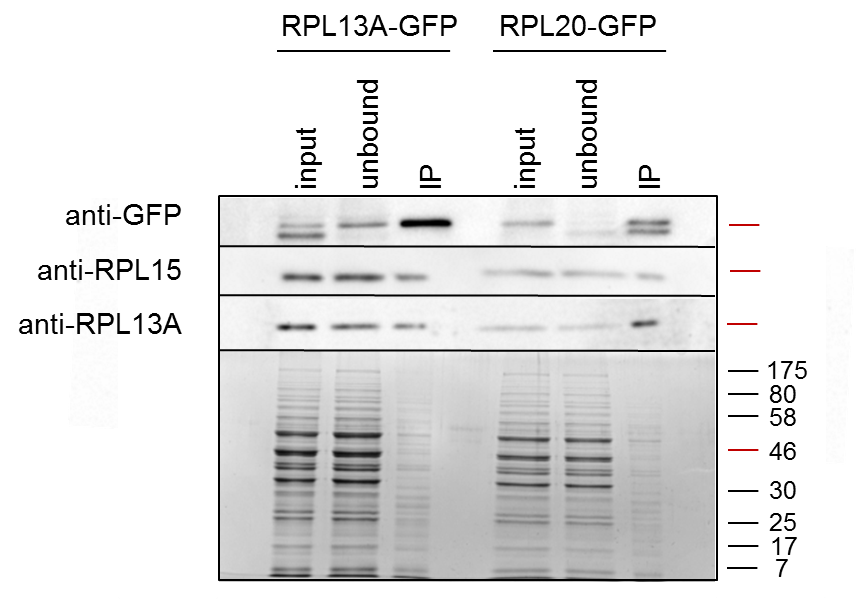

Supplement: S2 Fig — The ribosomal proteins, RPL15, Rpl13A and are detected by western blot in the immune-precipitated fractions. Coomassie brilliant blue stained gel shows total proteins in input, unbound and elution (IP) fractions. An immunoprecipitation of RPL20-GFP under identical conditions is shown as a comparison. Whole cell extracts were prepared from mid exponential cultures using a FastPrep-24 Instrument (MP Biomedicals, Santa Ana, CA, USA), antibodies were from Abcam (Cambridge UK; ab98211; ab130992; ab90874), GFP-TRAP_A agarose beads were obtained from ChromoTek (Planegg-Martinsried, Germany) and used according to inductions by manufacturer. (TIF) [file pone.0167394.s002.tif]

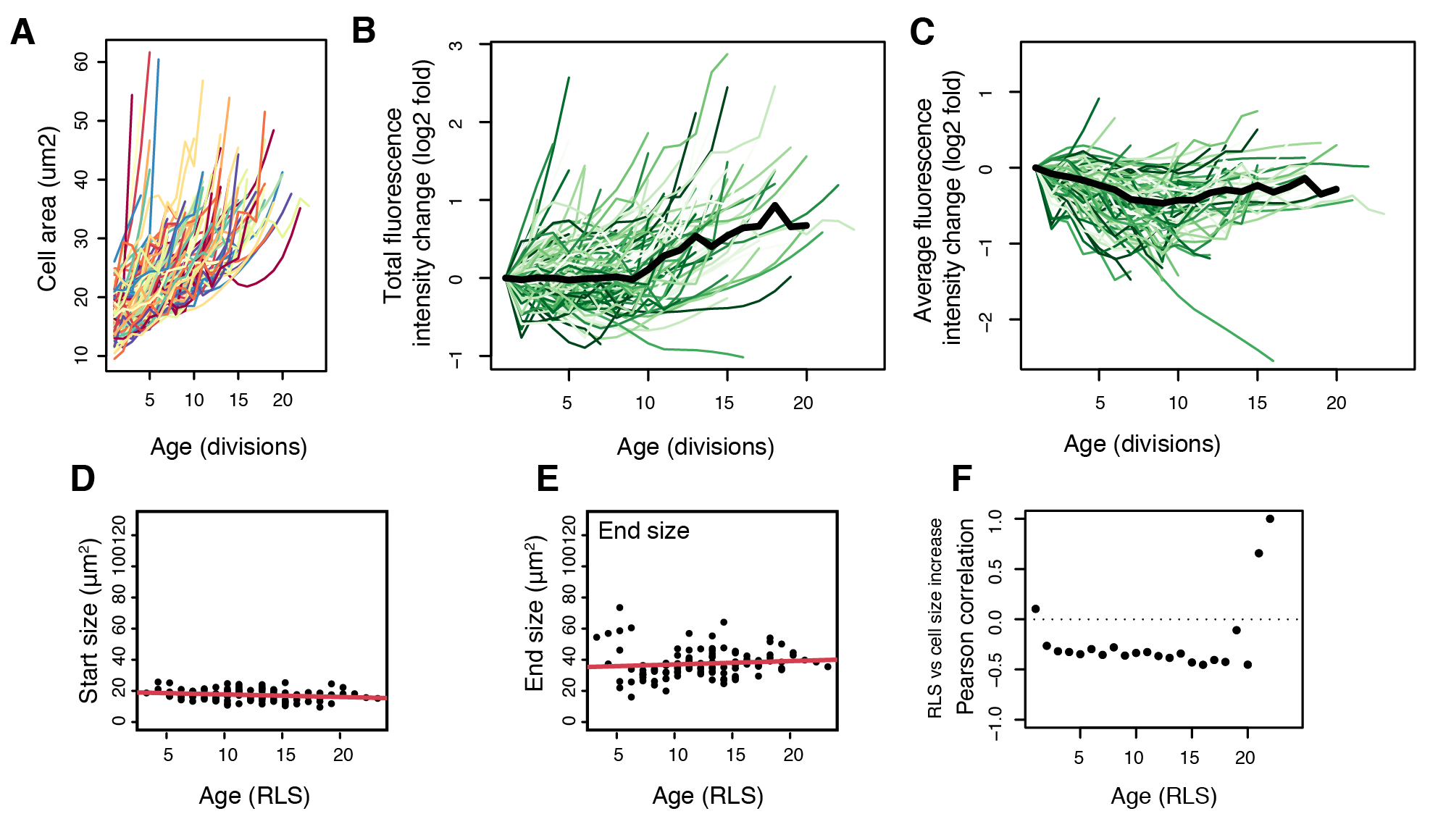

Supplement: S3 Fig — (A) Cell sizes same as Fig 2B but for the second dataset. (B) Same as Fig 4B but showing the fold change of the total fluorescence intensity of Rpl13A in the cell with age, indicating an increase in fold. Dark line is median. (C) Same as Fig 4C but showing the fold change of average intensity (concentration) of Rpl13A in the cell with age. Dark line is median. (DE) Same as Fig 2E and 2F, respectivelyA small negative correlation is found between starting cell size and lifespan (Pearson correlation -0.203 versus -0.047 in the first dataset). A small positive correlation is found between ending size and lifespan (Pearson correlation 0.101 versus 0.255 in the first dataset). (F) Same as 3B, showing a small negative correlation of lifespan compared to cell size increase. (TIF) [file pone.0167394.s003.tif]

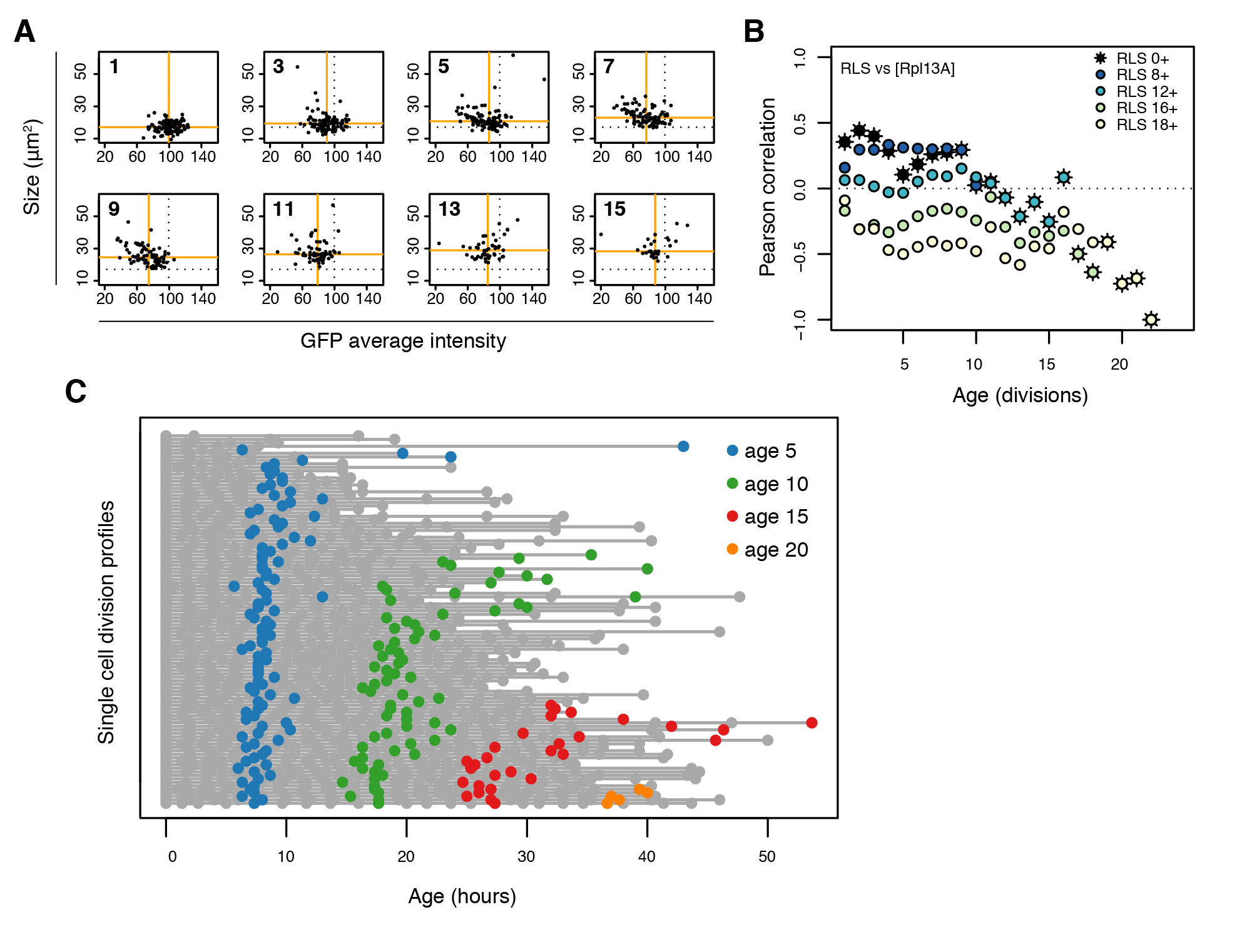

Supplement: S4 Fig — (A) Illustrates relationship between ribosome concentration and cell size throughout aging as does Fig 5B but for additional ages (numbers in top left of panels). (B) Same as Fig 5A (black dots, with stars), but illustrating the correlation of ribosome concentration to lifespan, when only considering cells in the population that lived at least to age 8, 12, 16, or 18. Illustrates that as the short lived cells are progressively removed from the analysis, the negative correlation of ribosome to lifespan becomes more prominent. (C) Same as Fig 6C but for second dataset. Confirms that longer-living cells (RLS, bottom of graph) divide more rapidly, i.e. reaching age 5 (blue dots) sooner in time than shorter-lived cells (RLS, top of graph). (TIF) [file pone.0167394.s004.tif]
